# Successful Treatment of Posttransplant Monoclonal Gammopathy-associated C3 Glomerulopathy With Plasma Cell Clone-directed Therapy

**DOI:** 10.1097/TXD.0000000000001616

**Published:** 2024-04-09

**Authors:** Ayman Al Jurdi, Abraham Cohen Bucay, Leonardo V. Riella, Andrew J. Yee, Cherif Abdelmalek, Veronica Klepeis, Shoko Kimura, Kassem Safa

**Affiliations:** 1 Division of Nephrology, Massachusetts General Hospital, Boston, MA.; 2 Division of Hematology and Oncology, Massachusetts General Hospital, Boston, MA.; 3 Division of Hematology and Oncology, Southern New Hampshire Health, Nashua, NH.; 4 Department of Pathology, Massachusetts General Hospital, Boston, MA.; 5 Division of Transplant Surgery, Massachusetts General Hospital, Boston, MA.

## Abstract

**Figure:**
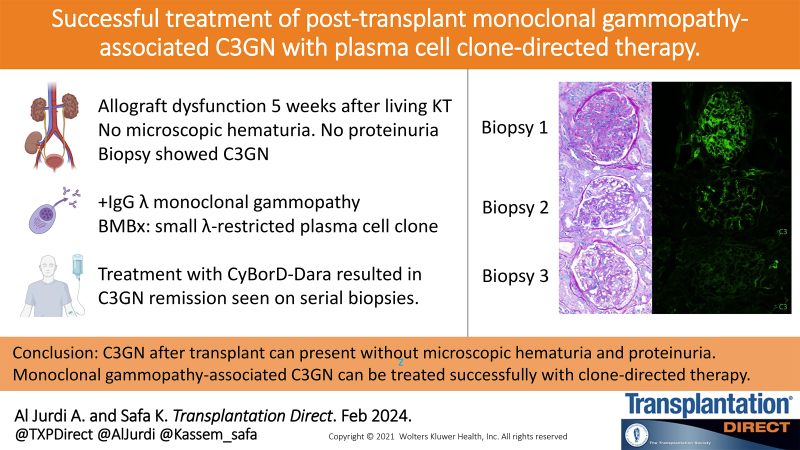

## INTRODUCTION

C3 glomerulonephritis (C3GN) results from activation of the alternative complement pathway (ACP) frequently because of genetic mutations or autoantibodies targeting regulators of the pathway.^1^ C3GN recurrence after kidney transplant is common and is associated with a high risk of allograft loss.^2,3^ Limited data exist on the treatment of C3GN after kidney transplantation.^3,4^ In this case report, we highlight a case of early C3GN after living unrelated kidney transplantation associated with an IgG λ monoclonal gammopathy that was treated successfully with plasma cell clone-directed therapy.

## CASE DESCRIPTION

**FIGURE 1.**
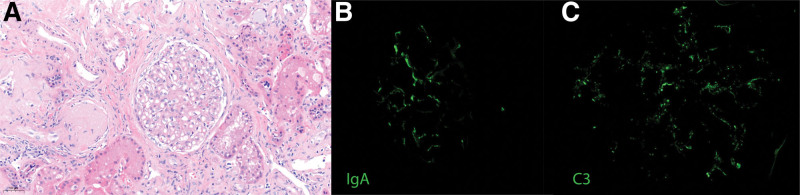
Native kidney biopsy 5 y before kidney transplantation. A, Hematoxylin and eosin staining showing moderate to severe arteriolosclerosis and a glomerulus with mild mesangial expansion and focally increased mesangial cellularity (40×). Immunofluorescence showing (B) 2+ IgA and (C) 1–2+ C_3_ granular mesangial staining.

**FIGURE 2.**
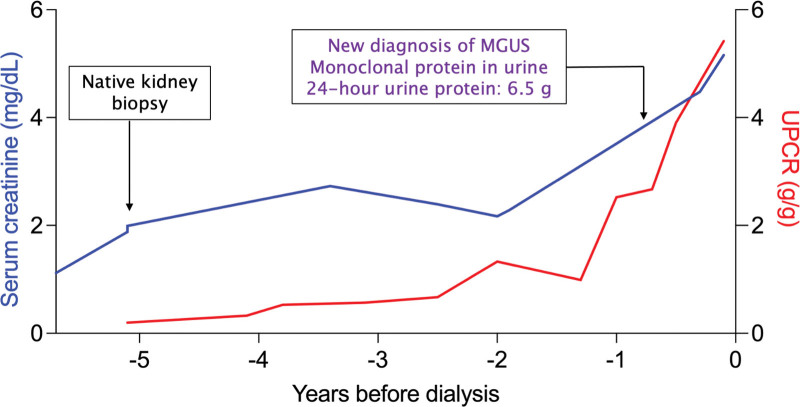
Pretransplant course. Predialysis trend of serum creatinine (blue) and UPCR (red). UPCR, urine protein-to-creatinine ratio.

A 63-y-old man underwent a kidney allograft biopsy 5 wk after transplantation for kidney allograft dysfunction. He had end-stage kidney disease secondary to vascular disease and possible IgA nephropathy based on a native kidney biopsy from 5 y prior (Figure 1A–C). His chronic kidney disease had progressed, and hemodialysis was initiated 7 mo prior (Figure 2). He had received a living unrelated kidney transplant 5 wk earlier with rabbit antithymocyte globulin induction (4.5 mg/kg) and maintenance immunosuppression with tacrolimus (trough levels at 8–10 ng/mL), mycophenolate sodium 540 mg twice daily, and prednisone 5 mg once daily. He did not have antihuman leukocyte antigen antibodies pretransplantation. His course was complicated by delayed graft function requiring only 1 hemodialysis session. However, 5 wk after transplantation, his serum creatinine remained at 2.9 mg/dL so a kidney allograft biopsy was performed.

His medical history is notable for a 0.32 g/dL IgG λ monoclonal gammopathy diagnosed 1 y before transplant on routine pretransplant screening. Bone marrow biopsy at the time showed a normocellular marrow with mixed hematopoiesis and no morphologic evidence of plasma cell neoplasm. Congo red stain was negative for amyloid. Flow cytometry of the aspirate showed a small lambda-restricted plasma cell population. Conventional skeletal survey did not show lytic bone disease, and positron emission tomography-computed tomography did not show fluorodeoxyglucose avid bone involvement. This was interpreted as monoclonal gammopathy of undetermined significance for which annual monitoring was recommended.

**FIGURE 3.**
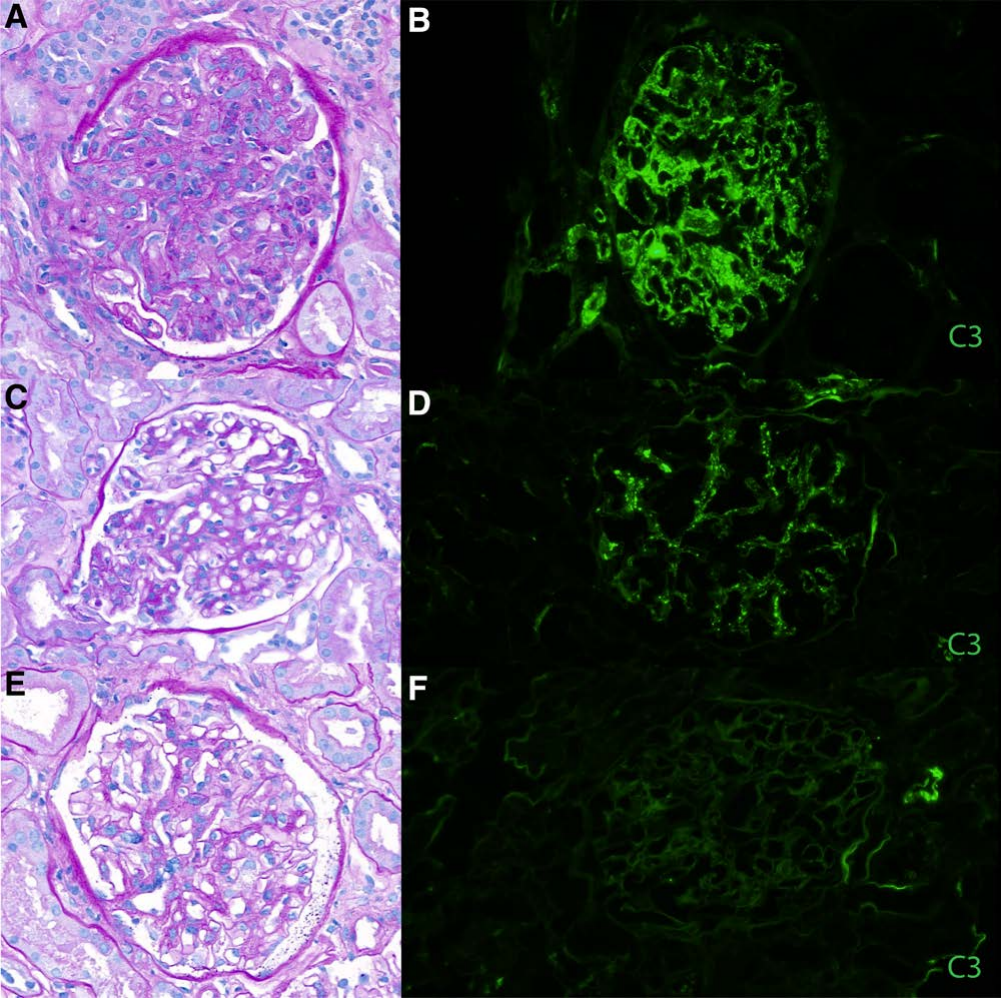
Kidney allograft biopsies. A, Hematoxylin and periodic acid-Schiff (H and PAS, 40×) staining of the first kidney allograft biopsy 1 mo after transplantation showing glomerulitis with a membranoproliferative pattern of injury and (B) granular 3+ C3 staining along the glomerular basement membrane. C, PAS (40X) staining of the second kidney allograft biopsy showing improving glomerulitis and (D) C3 deposits that decreased in number. E, PAS (40×) staining of the third kidney allograft biopsy showing resolution of glomerulitis and (F) negative C3 deposits staining. PAS, periodic acid-Schiff.

Before the kidney allograft biopsy, workup with urinalysis showed no blood or protein, urine protein-to-creatinine ratio (UPCR) was 0.15 g/g, BK viral load was undetectable, and antihuman leukocyte antigen antibody testing was negative. Pathology showed C3 glomerulonephritis (C3GN, Figure 3A and B) with a membranoproliferative pattern of injury and <5% interstitial fibrosis/tubular atrophy (IF/TA). Electron microscopy showed segmental podocyte foot process effacement and occasional amorphous deposits located in a mainly mesangial and focally subendothelial distribution This was thought to be less likely donor-derived, and the C3GN was thought to be either recurrent or de novo. The early posttransplant time was suspicious for recurrent C3GN so the patient’s pretransplant history was investigated further.

His native kidney biopsy slides were obtained and reviewed by our renal pathologists, which confirmed the findings of vascular disease and mesangial IgA deposits (IgA 2+ and C_3_ 1–2+ without evidence of endocapillary hypercellularity). At the time of his native kidney biopsy (5 y before transplant), serum creatinine was 2 mg/dL, UPCR was 0.2–0.3 g/g, and he had microscopic hematuria. At the time, he had a negative antineutrophil cytoplasmic antibody and normal C_3_/C_4_ levels. His chronic kidney disease progressed very slowly over the following 4 y, with serum creatinine increasing from 2.0 to 2.5 mg/dL. However, it was noted that he developed new nephrotic-range proteinuria (>6 g/g) a year before transplant, similar to the time of diagnosis of the monoclonal gammopathy. Serum creatinine then increased from 2.5 to 5 mg/dL during a 9-mo period. A repeat biopsy was not performed as the kidney disease was deemed end-stage and hemodialysis was started. This information was suggestive of the development of a new glomerular disease pretransplant, which further supported the theory that the C3GN was more likely to be recurrent than de novo.

Given the current pathologic findings, he had a genetic and functional ACP evaluation sent. Serum protein electrophoresis showed a 0.19 g/dL IgG-λ monoclonal gammopathy, free kappa light chain 17 mg/L, and free lambda 11.9 mg/L. Urine protein electrophoresis did show a monoclonal protein. Autoimmune disease workup showed a positive antinuclear antibody (1:160), normal antineutrophil cytoplasmic antibody, normal C_3_, high C_4_, and negative infectious workup. Although awaiting ACP testing results, and given the striking biopsy findings, intravenous steroids, eculizumab, and appropriate antimicrobial prophylaxis were started with improvement in serum creatinine. He was then discharged home, with a plan for further follow-up.

ACP functional evaluation showed evidence of complement dysregulation at the C3 and C5 convertase levels as indicated by the high fluid phase activity assay and high Bb, low properdin, and high soluble C5b-9. ACP genetic evaluation did not show any pathogenic mutations but showed 2 variants of unknown significance. Mutations identified included (1) a heterozygous deletion of CFHR3-CFHR1, which is present in 19.8% of European Americans and is unlikely to be clinically relevant^5^ and (2) a C3 heterozygous gene mutation: NM_000064.4:c.4506G>A, p.Leu1502 = (19:6679458:C > T), which can result in low C3 levels, the functional effect of it is not known.

Given his older age, the inconclusive functional/genetic ACP evaluation, and the presence of a monoclonal gammopathy, it was concluded that the C3GN was most likely due to monoclonal paraprotein-induced ACP activation. Given the aggressiveness of the disease, it was decided to pursue treatment with plasma cell clone-directed therapy: daratumumab with cyclophosphamide, bortezomib, and dexamethasone (dara-CyBorD) for 6 mo followed by maintenance daratumumab, similar to the ANDROMEDA regimen used in AL amyloidosis.^6^ Eculizumab and mycophenolate were stopped, and the chemotherapy regimen was initiated. He had received 2 doses of eculizumab 900 mg 1 wk apart at that point.

His course was complicated by acute kidney injury with a serum creatinine increase to 2.8 mg/dL. Allograft ultrasonography showed no evidence of obstruction, urinalysis was unremarkable, and UPCR was 0.07 g/g. Repeat serum protein electrophoresis showed a decreased IgG λ M spike (0.08 g/dL) consistent with improving paraproteinemia, and a 0.04 IgG M spike consistent with circulating daratumumab. A repeat soluble C5b-9 was sent, and repeat allograft biopsy was performed. Given the persistent allograft dysfunction and suspicion of ongoing C3GN, he was given a dose of eculizumab 900 mg while awaiting the final biopsy results.

**FIGURE 4.**
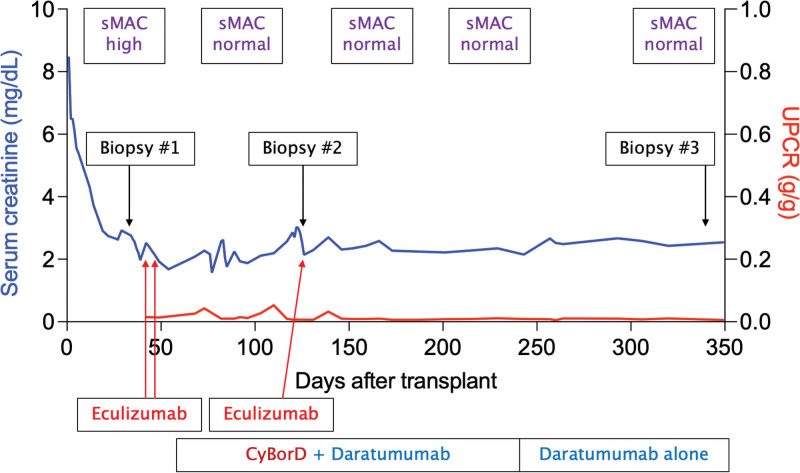
Treatment course after kidney transplantation. Posttransplant course and treatment, including serum creatinine levels, UPCR, timing of medication administration and soluble MAC levels. Each red arrow refers to a 900-mg dose of eculizumab given. MAC, membrane attack complex; UPCR, urine protein-to-creatinine ratio.

The second allograft biopsy showed improving C3GN and <5% IF/TA (Figure 3C and D). The soluble C5b-9 level (sent before eculizumab) returned normal. Given the stable kidney allograft function, improving glomerulitis on biopsy, decreasing level of IgG λ M spike, and normalization of the soluble C5b-9 level with clone-directed therapy, the patient did not receive any additional eculizumab. He completed 6 mo of dara-CyBorD followed by maintenance daratumumab monotherapy. His kidney allograft function improved and remained stable (serum creatinine: 2.1–2.5 mg/dL) and he had no proteinuria. One-year posttransplant, he underwent a surveillance allograft biopsy. This showed complete resolution of C3GN, mild BK polyomavirus-associated nephropathy, and <5% IF/TA (Figure 3E and F). His tacrolimus dose was reduced (aiming for a trough level of 4–6 ng/mL) with improvement in BK viremia. His course and treatment are summarized in Figure 4.

## DISCUSSION

This case highlights several key points regarding prekidney transplant evaluation and posttransplant C3GN treatment. First, it is of paramount importance to determine the cause of native kidney disease in individuals before kidney transplantation as that can have important implications about both risks of native disease recurrence and potential prevention strategies. In this case, the presence of a prior kidney biopsy likely biased against further evaluation for the new nephrotic-range proteinuria appearing several years after the original kidney biopsy and the associated accelerated decline in kidney function. In our opinion, pretransplant evaluation of the new nephrotic-range proteinuria, including a repeat kidney biopsy at the time, would have likely revealed the diagnosis and prompted treatment that may have slowed the progression of C3GN and possibly reduced the risk of posttransplant recurrence.

The second point is that although C3GN classically presents with microscopic hematuria and proteinuria in native kidneys,^2^ it can present with no microscopic hematuria, and with minimal or no proteinuria posttransplant as highlighted in our case and others,^2^ possibly related to the antiproteinuric effects of calcineurin inhibitors.^7^ Therefore, in individuals at risk for recurrent C3GN, lack of proteinuria and hematuria should not be considered a reliable marker for lack of recurrence. In individuals with C3GN and elevated soluble C5b-9 levels at C3GN diagnosis, which constitute a majority of individuals with C3GN and monoclonal gammopathy,^8,9^ a decrease in soluble C5b-9 levels to normal levels may be a useful marker of improving disease activity. Indeed, the level normalized after chemotherapy and correlated with improving disease activity on the second and third biopsies.

Finally, in individuals with C3GN, determining the underlying cause of ACP activation is critical to determining the risk of recurrence and the optimal treatment regimen.^2^ ACP genetic and functional testing is crucial. It is also important to evaluate for underlying monoclonal gammopathies, especially in older individuals with C3GN, as shown by Zand et al.^10^ This association is supported by laboratory evidence of monoclonal light chains or immunoglobulins directly activating the ACP^9,11^ and by improvement in C3GN in individuals with a concomitant monoclonal gammopathy who receive clone-directed therapy.^8,10,12^ It is important to note that C3GN in the setting of monoclonal gammopathy is considered to be a rare subtype of monoclonal gammopathy of renal significance where no monoclonal protein deposits are seen in the kidney.^13^ In our case, the histopathological improvement in C3GN was most likely due to the clone-directed therapy as opposed to eculizumab, given that eculizumab was only given briefly and by the improvement in C3 deposition, which would not be explained by terminal complement blockade. Since C3GN involves activation of the proximal complement pathway, newer drugs targeting the ACP more proximally, such as iptacopan, are currently being evaluated in clinical trials (NCT04817618) for C3GN treatment.
